# Local Tandem Repeat Expansion in *Xist* RNA as a Model for the Functionalisation of ncRNA

**DOI:** 10.3390/ncrna4040028

**Published:** 2018-10-19

**Authors:** Neil Brockdorff

**Affiliations:** Department of Biochemistry, University of Oxford, Oxford OX1 3QU, UK; neil.brockdorff@bioch.ox.ac.uk

**Keywords:** X inactivation, Xist, lncRNA, tandem repeat

## Abstract

*Xist*, the master regulator of the X chromosome inactivation in mammals, is a 17 kb lncRNA that acts *in cis* to silence the majority of genes along the chromosome from which it is transcribed. The two key processes required for *Xist* RNA function, localisation *in cis* and recruitment of silencing factors, are genetically separable, at least in part. Recent studies have identified *Xist* RNA sequences and associated RNA-binding proteins (RBPs) that are important for these processes. Notably, several of the key *Xist* RNA elements correspond to local tandem repeats. In this review, I use examples to illustrate different modes whereby tandem repeat amplification has been exploited to allow orthodox RBPs to confer new functions for *Xist*-mediated chromosome inactivation. I further discuss the potential generality of tandem repeat expansion in the evolution of functional long non-coding RNAs (lncRNAs).

## 1. Introduction

The long non-coding RNA (lncRNA) *Xist* mediates X chromosome inactivation (X inactivation), the process that, in mammals, equalises levels of X-linked gene expression in XX female relative to XY male cells [1,2,3,4,5]. *Xist* RNA is expressed from the inactive chromosome-elect at the onset of X inactivation during early embryogenesis and then localises to a subnuclear domain corresponding to the X chromosome nuclear territory [4,6]. Localised *Xist* RNA induces chromosome inactivation by recruiting factors that modify underlying chromatin and repress gene activity [7]. The repressive chromatin state on the inactive X chromosome (Xi), once established, is maintained through subsequent cell divisions in development and adult life.

A deletion analysis using *Xist* transgenes suggested that the localisation of *Xist* RNA and *Xist*-mediated silencing are separable processes [8]. Further support for separation of gene-silencing and localisation of *Xist* RNA has come from recent studies identifying RNA binding proteins (RBPs) that interact with *Xist* RNA, and that function principally in *Xist* RNA localisation (hnRNPU and Ciz1) [9,10,11], or *Xist*-mediated gene silencing (Spen, Rbm15/m6A-methyltransferase complex, LBR, and hnRNPK) [12,13,14,15,16,17].

**Figure 1:**
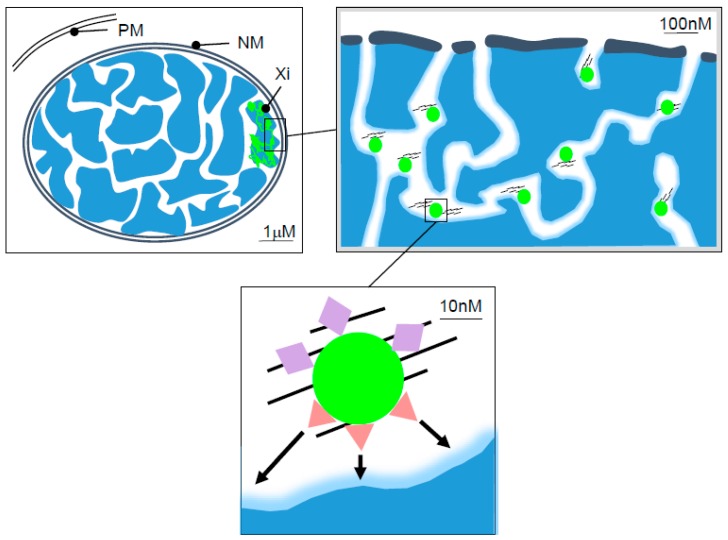
*Xist* RNA in the interphase nucleus. The schematic represents cross-sections of the nucleus illustrating the deduced relationship of *Xist* RNA (green) relative to the inactive X chromosome (Xi), interphase chromatin (blue), and interchromatin channels at different scales. The green circles represent single *Xist* RNA molecules. PM and NM denote the plasma- and nuclear-membrane respectively. The black lines represent the nuclear matrix proteins. Coloured shapes indicate the *Xist* RNA-associated proteins linked to tethering *Xist* RNA to the nuclear matrix (lilac diamond) or *Xist*-mediated chromatin silencing (red triangles). Arrows indicate the activity of the silencing factors towards proximal chromatin sites.

Further studies on *Xist* RBPs and the elements to which they bind have provided important advances in our understanding of *Xist* RNA localisation and *Xist*-mediated silencing. Several observations have led to the idea that *Xist* RNA is anchored to the Xi territory through the interaction with the nuclear matrix: Thus, *Xist* RNA domains are retained in cells following nuclear matrix extraction, a procedure which yields nuclei from which the bulk of chromatin and soluble proteins are removed [18]. Additionally, the knockdown/knockout of the *Xist* localization factors hnRNPU and Ciz1, both of which have been characterised as components of the insoluble nuclear matrix, leads to the delocalisation of *Xist* RNA to sites throughout the nucleoplasm [9,10,11]. Finally, analysis by 3D-SIM, a method for super-resolution light microscopy, has revealed that *Xist* RNA colocalises with hnRNPU and Ciz1 within channel networks that pervade interphase chromosome territories, including on Xi [19,20]. Extrapolating from this idea, it can be inferred that anchored *Xist* RNA molecules function to nucleate chromosome silencing through the recruitment/enrichment of *Xist* silencing factors (Figure 1).

**Figure 2:**
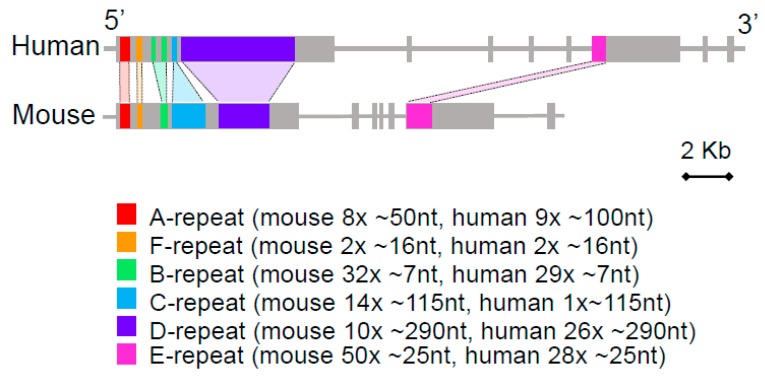
Local tandem repeats in *Xist* RNA. The schematic illustrates the intron/exon structure of human and mouse *Xist* genes with conserved tandem repeat blocks indicated in different colours. The key indicates the label, approximate copy number, and monomer length for each repeat block based on the mouse and human *Xist/XIST* RNA sequence.

Super-resolution microscopy experiments have further revealed that *Xist* RNA domains are comprised of around 100–200 foci, representing individual *Xist* ribonucleoprotein particles (RNPs) [20,21]. An important challenge is to understand how this relatively small number of *Xist* RNPs induces gene silencing across an entire chromosome, comprising some 150 Mb of DNA and around 1000 genes. One factor that may contribute is signal amplification through local tandemly repeated RBP binding sites within single *Xist* RNA molecules. Specifically, the sequence analysis of *Xist* RNA in mouse, human and other species has revealed that a significant proportion of the primary RNA sequence is comprised of blocks of local tandemly repeated elements, designated repeats A–F (Figure 2). Several of these blocks are conserved in *Xist* from different mammalian species [2,4,22], and moreover, in many cases, they have been shown to bind RBPs that play a role in *Xist* RNA localisation and *Xist*-mediated silencing [10,11,13,15,17]. In this review, I will discuss the emerging evidence regarding how *Xist* tandem repeats contribute to function, and in addition, the wider implications for these findings in understanding the evolution of functional lncRNAs.

## 2. Interaction of the RBPs Spen and Rbm15 with the *Xist* A-Repeat Element

The *Xist* A-repeat, located at the 5′ end of *Xist* RNA, comprises of 7–8 copies of a 24 nt GC-rich core sequence separated by uridine tracts of variable length [2,8]. The deletion of the A-repeat from full-length *Xist* RNA largely ablates *Xist*-mediated silencing, but does not grossly affect *Xist* RNA localisation [8]. However, in the context of a truncated *Xist* RNA transgene spanning the first 4 kb of sequence, deletion of the A-repeat does lead to loss of *Xist* RNA localisation [8,17], attesting to functional redundancy of localisation elements in full-length *Xist* RNA. Given the essential role of the A-repeats, there has been considerable interest in defining the RBPs that interact with this element. To this end recent proteomic and genetic screening strategies have identified that the RBP Spen is recruited to the A-repeat and functions as a critical factor in *Xist*-mediated silencing [12,13,14,15], recruiting the NCoR-HDAC3 complex to deacetylate chromatin at target sites [12]. A closely related RBP, Rbm15, that has been implicated in *Xist*-mediated silencing has also been shown to bind to the A-repeat region [14,23]. Rbm15 interacts with the multiprotein enzyme complex that catalyses the N6-adenosine (m6A) methylation of RNA, and it is thought that Rbm15 functions target m6A to sites across *Xist* RNA to facilitate the silencing function [14,23]. The precise mechanism of the Rbm15/m6A function in *Xist* silencing is not well defined but is thought to involve the m6A reader protein, YTHDC1 [23].

An interesting speculation is that the evolutionary expansion of A-repeat monomers has increased the number of Spen molecules that can concurrently bind to a single *Xist* RNA molecule, thereby enhancing the silencing activity. Spen is a large protein, some 3600 amino acids, which includes four closely spaced RNA binding (RRM) domains at the N-terminus, and a Spen Paralogue and Orthologue C-terminal (SPOC) domain located at the C-terminus [24,25,26,27]. The SPOC domain mediates the interaction with the NCoR-HDAC3 complex [26]. In vitro studies using a truncated N-terminal Spen protein spanning RRMs 2–4 have determined that binding to the A-repeat monomer occurs with a 1:2 stoichiometry [28]. This conclusion is based on the observation that the overall A-repeat structure is comprised of pairs of monomers, and the finding that Spen RRM 2–4 binds to single-stranded regions, determined by in vitro CLiP (UV cross-link analysis). Thus, in relation to signal amplification, each *Xist* RNA molecule has the potential to bind 3–4 Spen molecules, a moderate enhancement. It is interesting to note that the complementation of the A-repeat deletion can be achieved using a synthetic X7.5 A-repeat consensus sequence, but not with an X5.5 A-repeat [8]. Whether or not the A-repeat is bound by multiple Spen molecules contemporaneously in vivo remains to be determined. Thus, the large size of the Spen protein may sterically hinder nearby binding sites. Additionally, it is likely that Rbm15, which has a very similar configuration of N-terminal RRM domains to that of Spen [29], competes for A-repeat binding sites.

**Figure 3:**
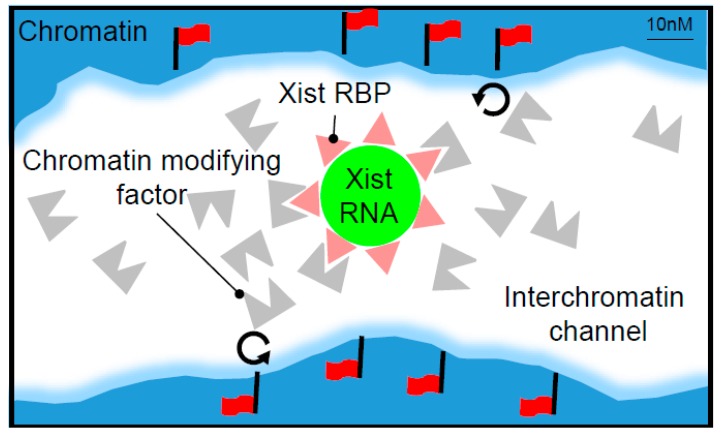
*Xist* ribonucleoprotein particles (RNPs) generate local concentrations of silencing factors within interchromatin channels. The schematic illustrating a model for how *Xist* RNA bound RNA binding proteins (RBPs) (red triangles) can function to generate a local concentration of chromatin-modifying factors (grey shape). Multiple RBP molecules that are strongly bound to a tandemly repeated element on the anchored *Xist* RNA molecule (green) interact weakly/transiently with the chromatin-modifying factor such that the local concentration of unbound molecules increases within the interchromatin channel. The unbound chromatin-modifying factor can then act at the available chromatin sites (circular arrows) indiscriminately within a zone surrounding the *Xist* RNP, resulting in a widespread deposition or removal of specific chromatin modifications (red flags).

The interaction strength of Spen and Rbm15 with *Xist* RNA and with NCoR-HDAC3 or the m6A MTase complex, respectively, is also potentially important in relation to signal amplification. Thus, relatively weak and, hence, dynamic interactions could contribute to signal amplification by creating a high local concentration of effector proteins in interchromatin spaces (Figure 3). In the case of Spen, the in vitro analysis indicates that Spen RRMs bind A-repeat sequences with a high affinity, ~10 nM [28], suggesting a stable interaction. However, the interaction of Spen with NCoR, NCoR with HDAC3 could be relatively weak and dynamic, potentially underpinning the local concentration of the effectors to amplify function. A similar argument can be put forward for Rbm15 interactions with the A-repeat and m6A MTase complex respectively.

## 3. Recruitment of the Polycomb System by hnRNPK Bound to the *Xist* B/C-Repeat

A recent study has shown that the *Xist* RNA B-repeat, together with a short part of the C-repeat (Figure 2), directs the recruitment of Polycomb repressive complexes (PRC) 1 and 2, which also contribute to *Xist*-mediated gene silencing [17]. Briefly, the B-repeat is comprised of around 32 copies of a cytidine-rich heptameric sequence element which is bound by the RBP hnRNPK. hnRNPK is a multifunctional RBP characterised by 3 KH domains that mediate RNA binding, and an unstructured domain that links the protein to diverse interaction partners [30]. The recently identified hnRNPK interacting proteins include Pcgf3 and Pcgf5, homologous Polycomb proteins that function as core subunits of the non-canonical Pcgf3/5-PRC1 complex, responsible for initiating Polycomb recruitment by *Xist* RNA [17,31]. Pcgf3/5-PRC1 catalyses mono-ubiquitylation of lysine 119 in histone H2A (H2AK119ub1), a repressive chromatin modification which, in addition to contributing directly to gene repression in X inactivation, initiates a positive feedback cascade, recruiting other non-canonical PRC1 complexes, and a second complex, PRC2, which catalyses histone H3 lysine 27 tri-methylation (H3K27me3) [31]. The role of the C-repeat region in this pathway has not been clearly defined but it is also bound by hnRNPK, albeit at a low level [32].

As argued above for Spen, the recruitment of multiple hnRNPK molecules by B/C-repeat monomers may serve to enhance *Xist*-mediated chromosome silencing. Each KH domain in hnRNPK binds to separate 6–7 nt C-rich tracts in a cooperative manner [33], and it follows that up to 10 hnRNPK molecules could bind to the B-repeat element of a single *Xist* molecule. This has not yet been tested either in vitro or in vivo, and as argued above, factors such as steric hindrance or competition with other RBPs may influence the occupancy of the B-repeat. Similar to Spen, hnRNPK binds RNA with a high affinity, in the low nanomolar range [34], whereas the interaction of hnRNPK with Pcgf3/5 appears to be relatively weak, as judged from the low stoichiometry of hnRNPK in mass spectrometry analysis using Pcgf3/5 as bait (unpublished work). Thus, in vivo, hnRNPK stably bound to the *Xist* B-repeat may function to amplify the local concentration of the Pcfg3/5-PRC1 effector complex within interchromatin spaces of Xi, with consequential widespread modification of the underlying chromatin, as depicted in Figure 3. Consistent with this idea, Polycomb mediated H3K27me3 is evenly distributed over Xi chromatin [19,35,36,37], contrasting with conventional Polycomb sites where the deposition is limited to relatively discrete elements at the promoters of target genes [38].

A further contribution to signal amplification in this example comes from the positive feedback between different Polycomb complexes and their respective histone modifications, as detailed above. Overall, the successive amplification steps in the B-repeat-mediated recruitment of the Polycomb system provide a compelling rationale for how relatively small numbers of *Xist* RNA molecules establish chromatin modification and gene silencing across an entire chromosome.

## 4. Ciz1, an Anchoring Factor, Is Recruited by the *Xist* E-Repeat

As noted above, the *Xist* RBP Ciz1 facilitates the anchoring of *Xist* RNA to the Xi territory [10,11]. This function is only evident in specific somatic cell types, fibroblasts, and B- and T-lymphocytes, where Ciz1 loss of function causes the dispersal of *Xist* RNA particles throughout the nucleoplasm [10]. As Ciz1 colocalises with *Xist* RNA in all cell types, the cell type specificity of the phenotype likely reflects redundancy in the mechanism for *Xist* RNA localisation. A known alternative pathway involves hnRNPU, the depletion of which results in *Xist* RNA dispersal in many cell types, including embryonic stem cells [9,12,13,39,40]. There is evidence that hnRNPU, which has an RRM domain, interacts directly with *Xist* RNA, although an analysis by CLiP indicates that the binding sites are broadly distributed throughout the transcripts, not centred on any specific unique or local tandem repeat element [32].

Ciz1 was originally identified as a protein that interacts with the cell cycle inhibitor CDKN1A and was subsequently shown to have a role in initiating DNA replication, and in cell cycle progression at the G1/S checkpoint [41]. Biochemical fractionation experiments found that Ciz1 is enriched in the nuclear matrix compartment [42]. A role as an RBP was suggested due to the presence of putative RNA binding zinc-finger domains, including a matrin-like zinc-finger. Evidence for an interaction with a known RNA, specifically *Xist*, came only recently [10,11]. Ciz1 binding was mapped to the *Xist* E-repeat, comprising of an approximately 20–25 nt sequence element tandemly repeated to approximately 50 copies (Figure 2). Whilst the sequence requirements and stoichiometry of Ciz1 binding to the E-repeat remain to be determined, the strong enrichment of Ciz1 in association with *Xist* RNPs compared to elsewhere in the nucleus, as determined by super-resolution microscopy [10], indicates that there are indeed multiple Ciz1 molecules bound to each *Xist* RNA molecule. The precise relationship of Ciz1 and hnRNPU in *Xist* RNA localisation is not clear, but the hnRNPU knockout in fibroblasts does result in the dispersal of *Xist* particles that continue to be associated with Ciz1 [11]. This latter observation suggests that hnRNPU is required for Ciz1 to confer *Xist* RNA anchoring, either due to a direct interaction or indirectly, for example playing a role in the formation of the nuclear matrix, the substrate for anchoring. The latter idea is supported by recent evidence that hnRNPU regulates interphase chromosome structure via oligomerization with chromatin-associated RNAs [43].

**Figure 4:**
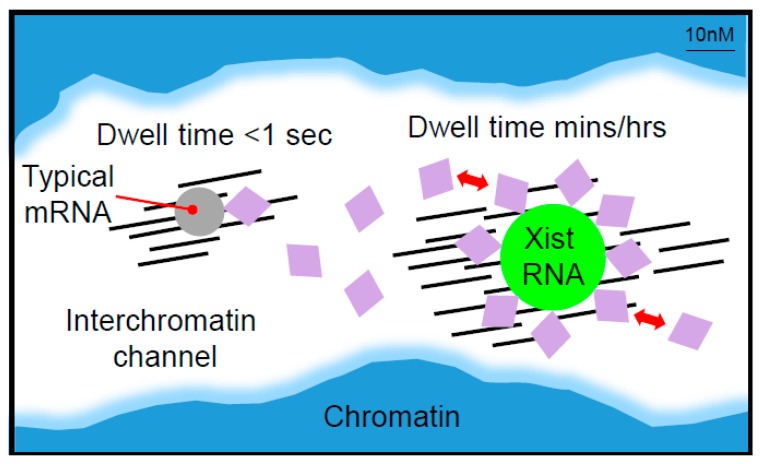
The amplification of RBP binding sites as a driver for *Xist* RNP anchoring. A schematic illustrating a model for how the amplification of RBP-binding sites on *Xist* RNA facilitates RNA anchoring. For a typical messenger RNA (mRNA) (grey circle), an RBP (lilac diamond) that interacts transiently with nuclear matrix proteins (black lines) immobilises the mRNA in interchromatin channels for a short time (hypothetical dwell time <1 s). For *Xist* RNA (green circle), amplification of the number of binding sites for the RBP increases the dwell time hypothetically up to minutes or even hours in a manner proportional to the local concentration of the RBP, the interaction strength with *Xist* RNA, and the number of binding sites.

It is interesting to consider that the association of multiple Ciz1 molecules with the E-repeat underpins its role in anchoring *Xist* RNA particles. Specifically, if we assume that Ciz1 is relatively immobile as a consequence of its interaction with the nuclear matrix, but interacts with typical target RNAs that have a single or a few Ciz1 binding sites dynamically/transiently, then an RNA that has evolved multiple recognition motifs would be predicted to have a significantly increased dwell/retention time, as illustrated in Figure 4. This idea is conceptually distinct from *Xist* RNA repeats serving to increase the local concentration of the effector complexes proposed above in relation to the recruitment of Spen/Rbm15 and hnRNPK (Figure 3).

The *Xist* A-repeat and C-repeat have also been suggested to have a role in *Xist* RNA localisation [8,44,45] and, although the mechanistic basis for this is uncertain, it could also involve amplified binding sites for RBPs that enhance *Xist* RNA retention in the nuclear matrix fraction. This speculation is consistent with a redundancy in *Xist* RNA localisation pathways [8].

## 5. Local Tandem Repeat Amplification and the Evolution of *Xist* RNA Function

The accumulated evidence that local tandem repeats in *Xist* RNA are central to its role in X inactivation suggests a simple model for how *Xist* RNA evolved. Thus, tandem duplication of sequences that encompass the binding site for a common RBP in the archetypal *Xist* RNA, likely as a consequence of DNA replication errors, could have generated enhanced or modified RBP functions on which natural selection could act on. The theory of sex chromosome evolution implies that the dosage compensation evolved incrementally, with allelic repression of X-linked genes being selected initially within a relatively small region of the prototypic X chromosome (corresponding to the region in which the prototypic Y homologues become functionally compromised due to recombination suppression) [46]. With the progressive erosion of the prototypic Y chromosome, there would be a selective pressure for the X inactivation signal to spread further, eventually encompassing the entire X chromosome. Thus, the amplification of binding sites for RBPs that interact transiently with the nuclear matrix would be predicted to incrementally increase the dwell time of prototypic *Xist* RNPs, with the increased number of bound RBP molecules contributing to the strength of the interaction with the nuclear matrix. Evolution of strong interactions with the nuclear matrix would first serve to limit *Xist* RNP localisation *in cis*, and second, would be important in terms of modulating the range or distance of spread of *Xist* RNPs.

Using a similar argument, the amplification of binding sites for RBPs that generate a local concentration of silencing factors could be selected for on the basis of improved efficiency/completeness of gene dosage compensation. One possibility is that an RBP that binds to a single site in the prototypic *Xist* lncRNA may have a previously evolved interaction with a known transcriptional repressor. An example of this would be the RBP Spen, for which interaction with NCoR-HDAC3 is predicted to be evolutionarily conserved [26]. Alternatively, an RBP that binds to a unique site in the prototypic *Xist* lncRNA may acquire an interaction with a known repressor complex as a neofunctionality. This could be the case for hnRNPK which has roles in diverse aspects of RNA biogenesis [30], of which the recruitment of Pcgf3/5-PRC1 complexes to chromatin has only been documented to occur in the context of *Xist* RNA [17]. Thus, the amplification of hnRNPK-binding sites in *Xist* RNA may have been exploited to enhance a relatively weak interaction with the Pcgf3/5 Polycomb protein to a level sufficient to initiate the Polycomb cascade.

In the context of the above models, the long-term retention of *Xist* RNPs on the nuclear matrix is likely to be important in enabling the local concentration of silencing factors to reach a critical threshold. Accordingly, Spen/hnRNPK binding sites in other messenger RNAs (mRNAs) or lncRNAs may not lead to significant levels of chromatin modification *in cis*, i.e., in cases where the RNA is not anchored to the nuclear matrix for a significant time. Here it is interesting to note that hnRNPK binding to C-rich motifs has been implicated in retention of nuclear RNAs, including those arising from the Alu family of dispersed repeats [47].

## 6. A Role for Local Tandem Repeat Expansion in Other LncRNAs

Our growing appreciation of the importance of local tandem repeat expansion in functionalisation of *Xist* RNA leads to the question of how general this model may be in the evolution of functional lncRNA. There are indeed examples that illustrate that this mechanism may be more widely utilised. Thus, the lncRNA RNA on the Silent X (*Rsx*), which evolved as the master regulator of X inactivation in marsupial mammals entirely independently of *Xist*, is also characterised by the presence of large blocks of local tandem repeated sequences [48]. The sequence similarity of these elements and those present in *Xist* RNA is limited, and at present nothing is known about their functional importance or bound RBPs. However, close parallels in the X inactivation process in eutherian and metatherian mammals, notably chromosome-wide hypoacetylation of histones and recruitment of the Polycomb system, imply that commonalities could extend to key *Xist* silencing factors such as Spen and hnRNPK. There may also be overlaps in terms of the factors and mechanisms regulating *Rsx* RNA localisation, as, like *Xist* RNA, *Rsx* RNA localises strictly *in cis* on the X chromosome from which it is transcribed [48].

Whilst the focus of this review up to this point has been on local repeat sequences that are tandemly arranged, it should be noted that many of the arguments put forward apply equally well to local repeats that are dispersed within a given locus, referred to simply as local repeats. An example of this has emerged from recent studies on the lncRNA Firre/FIRRE [49,50]. Thus, a 156 bp local repeat termed RRD, present in 8 copies, is required for the nuclear localisation of FIRRE RNA, potentially through providing binding sites for the nuclear matrix protein hnRNPU. RRD is also present in mouse Firre RNA (16 copies). Although primary sequence conservation relative to primate RRD is only moderate (~60%), interaction with hnRNPU is conserved and knockdown experiments indicate hnRNPU-dependent nuclear retention both in human and mouse cells [50]. Another well-documented example of an lncRNA in which local repeat sequences play a functional role is NORAD, which regulates the genome stability by sequestering PUMILIO proteins that control mRNA stability [51,52].

Recent studies on the lncRNA Neat1, which is required to nucleate the formation of paraspeckles, a phase-separated membrane-less organelle [53], also point to the importance of the multivalent interaction of RBPs in lncRNA function. Specifically, the recruitment of proteins required to establish paraspeckles is mediated by several redundant modules present in the middle part of Neat1 lncRNA[54]. In this example, there is no available evidence for a locally repeated sequence common to independent modules. Possible explanations for this are that dissimilar sequences recruit the key RBPs required for paraspeckle assembly, or alternatively, that each independent module has a conserved secondary RNA structure that forms from apparently disparate primary sequences.

Whilst the amplification of RBP binding sites likely represents a key mechanism for the functionalisation of lncRNAs, it should be noted that the amplification of binding sites for other RNAs may also be an important evolutionary mechanism. Indeed, certain circular RNAs have evolved as molecular sponges, presenting multiple binding sites for specific miRNAs [55].

## 7. Summary

The expansion of local tandem repeats during the evolution of *Xist* RNA provides an instructive example of how lncRNAs may become functionalised through natural selection. Conversely, the analysis of local tandem repeat expansions, or for that matter dispersed local repeats, in lncRNA, together with the identification of RBPs that bind to them, could provide a useful approach towards understanding the function of specific lncRNAs. An interesting starting point would be to analyse lncRNAs associated with imprinted gene clusters as, at least in some cases, these likely function analogously to *Xist* to induce chromatin repression over contiguous genomic regions [56,57]. Similarly, lncRNAs transcribed from chromosomal regions comprising tandem DNA repeats, for example at telomeres [58], pericentric heterochromatin [59], and other repetitive regions [60], are interesting candidates. Determining which lncRNAs to focus on in terms of the potential for functionalisation through repeat expansion will be greatly facilitated by SEEKR, a recently described bioinformatics tool that analyses the k-mer (short motif) content within defined lncRNA sequences [61].
